# Internal and external factors drive vegetative-reproductive strategies and reveal plant developmental stage as a lever for precision grassland management

**DOI:** 10.1016/j.plaphe.2026.100254

**Published:** 2026-07-08

**Authors:** Yu-Wen Zhang, Jie-Yan Zhou, Zhao-Xia Guo, Lan Li, Fu-Jiang Hou

**Affiliations:** China-Kazakhstan Belt and Road Joint Laboratory on Grassland Ecological Restoration, Key Laboratory of Grassland Livestock Industry Innovation, Ministry of Agriculture and Rural Affairs, Engineering Technology Research Center for Ecological Restoration and Utilization of Degraded Grassland in Northwest China, National Forestry and Grassland Administration, College of Pastoral Agriculture Science and Technology, Lanzhou University, Lanzhou, 730020, China

**Keywords:** Individual developmental stage, Phenotypic integration, Plant phenomics, Seasonal rotational grazing, Reproductive strategies

## Abstract

Advancing plant phenomics requires linking high-resolution phenotypic data to plant performance under environmental stresses like grazing. However, how intrinsic biological factors, specifically individual developmental stage, mediate phenotypic trade-offs in response to management remains poorly quantified. Using a phenomics approach on the dominant grass *Stipa bungeana* within a two-decade grazing experiment, we integrated multi-year, individual-level trait data to dissect the effects of grazing season (cold/warm), intensity (light/moderate/heavy), climate, and developmental stage (proxied by basal diameter) on vegetative and reproductive tiller phenomes. We show that warm-season grazing increased tiller production, whereas cold-season grazing simplified phenotypic correlation networks. Notably, developmental stage was the dominant driver of vegetative growth and individual biomass, while reproductive investment was primarily governed by external drivers (climate and grazing intensity). Path modeling revealed that developmental stage indirectly enhances sexual reproduction by fueling vegetative investment, jointly determining final biomass. This study suggests that individual developmental stage as a potential internal integrator of grazing signals, reshaping phenotypic architecture. Our findings provide a phenotype-driven framework for precision grassland management. By advocating for the monitoring of developmental stage composition, we bridge phenomics with sustainable practices, enabling dynamic grazing strategies that optimize the balance between productivity and ecosystem resilience.

## 1 Introduction

Grassland covers 22.8% of land (30.1 million km^2^), stores 155.02 Pg of soil carbon (0–30 cm depth), supports ∼850 million people, is vulnerable to human disturbance, and provides habitat for biodiversity, including many endemic plants and at-risk megafauna [1]. However, their ecological integrity and productivity are increasingly compromised by widespread degradation, largely attributed to unsustainable grazing practices [2,3]. A central challenge in sustainable rangeland management lies in understanding how dominant perennial grasses, the foundational architects of these ecosystems, balance persistence and productivity under recurrent disturbance [4]. While it is recognized that such species often employ a mixed strategy of clonal propagation and sexual reproduction to cope with grazing [[5], [6], [7], [8]], a critical knowledge gap persists. We lack a clear phenotype-level understanding of the putative pathways through which this reproductive trade-off is intrinsically modulated by an individual plant's developmental stage, and how this internal regulation ultimately determines biomass accumulation and population resilience.

The vast arid grasslands of northern China, covering some 280 million hectares, sustain the livelihoods of over 40 million people through pastoralism [9,10]. Yet, historical overgrazing under collective land tenure has triggered widespread degradation, undermining both ecological integrity and pastoral productivity [11]. In response, grassland privatization since the late 20th century aimed to mitigate the “tragedy of the commons,” yet it also introduced new challenges, such as habitat fragmentation and sedentarization, which in some cases exacerbated grassland loss [12,13]. To address this degradation, seasonal rotational grazing has emerged as a promising management approach [14,15]. Yet, a key knowledge gap remains: while management is often designed around livestock needs, it seldom accounts for how key grassland species, particularly facultative clonal plants like *Stipa*, respond to such disturbances. The balance between clonal propagation (ensuring local persistence) and sexual reproduction (enabling dispersal and genetic diversity) represents a fundamental life-history trade-off that shapes a plant's response to disturbance. While grazing intensity and season are known to influence this balance, the role of intrinsic plant traits, specifically individual developmental stage (a proxy for plant size and physiological age), as a mediator of these responses has been severely understudied.

The balance between clonal propagation and sexual reproduction represents a fundamental life-history trade-off in perennial grasses, directly influencing their persistence, dispersal, and genetic diversity [16]. Grazing influences this balance through multiple, interacting mechanisms: direct defoliation and trampling alter plant architecture and resource status, while the timing of nutrient return via livestock excreta can synchronize with or mismatch key phenological phases, thereby modulating reproductive investment [[17], [18], [19]]. Previous research has established that grazing intensity and season can shift biomass allocation between above- and belowground structures and between reproductive modes [8,20]. However, these responses are unlikely to be uniform across all individuals within a population. In tussock-forming species like *Stipa bungeana*, individuals of different sizes, a proxy for their developmental stage, occupy distinct physiological and competitive positions. Larger, more established tussocks may possess greater resource reserves but also face higher costs of self-shading, whereas smaller individuals might be more vulnerable to disturbance but also more plastic in their responses [21,22]. Despite this inherent variability, the role of individual developmental stage as a hypothesized internal filter that helps explain how external grazing signals are translated into phenotypic outcomes, specifically the trade-off between vegetative and reproductive tiller investment, has rarely been incorporated into a unified explanatory framework. Most studies aggregate data at the plot or population level, potentially obscuring these size-dependent strategies.

Therefore, to address these interconnected knowledge gaps, spanning theoretical ecology, phenotypic plasticity, and applied grassland management, we conducted a targeted investigation using *Stipa bungeana* as a model species within a long-term experimental platform. This perennial C_3_ grass dominates the typical steppes of northern China and exhibits a clear growth phenology concentrated between March and September, making it an ideal subject for studying trait-mediated responses to managed disturbance. By embedding a detailed, three-year phenotyping campaign within a unique two-decade grazing experiment, we move beyond population-level averages to explicitly examine how individual developmental stage (proxied by basal diameter) interacts with grazing season and intensity to govern phenotypic expression. We addressed the following research questions: (1) How do grazing season (cold vs. warm) and intensity differentially modify the morphology and biomass of vegetative and reproductive tillers? (2) Does individual developmental stage dominate the expression of vegetative traits and overall plant biomass, while reproductive investment remains more responsive to external drivers like climate and grazing pressure? (3) How does grazing alter the coordination and trade-offs among these key phenotypic traits? Corresponding to these questions, we hypothesized that: (H1) warm-season grazing would exert stronger stimulatory effects on tiller production than cold-season grazing; (H2) individual developmental stage would be the dominant driver of vegetative growth and biomass, whereas reproductive investment would be primarily governed by external drivers, with cold-season grazing simplifying trait correlation networks; and (H3) the divergent control of vegetative and reproductive modules by internal versus external factors would restructure phenotypic integration under grazing. By dissecting these statistical associations, we aim to provide a phenomically informed framework for grazing management. We propose that monitoring and accounting for the developmental stage composition of key plant populations could enable more dynamic and sustainable management strategies, ultimately helping to balance pastoral productivity with the long-term ecological resilience of these critical ecosystems.

## 2 Materials and methods

### 2.1 Study site description

This study was conducted in Huan County (106°50′ E, 37°7′ N), located in Gansu Province, China. The site lies within a transitional zone between the residual gully and desert regions of northern China and represents a significant pastoral region. The area is characterized by a temperate continental semi-arid monsoon climate, with a mean annual temperature of 8.0 °C and an average annual precipitation of 350 mm. The elevation of the study site is 1650 m above sea level. The grassland is classified as typical steppe [23], dominated by key species such as *Stipa bungeana*, *Artemisia capillaris*, and *Lespedeza davurica*. According to the Harmonized World Soil Database (HWSD), the predominant soil type is Calcaric Cambisols.

### 2.2 Overview of *Stipa bungeana*

**Fig. 1:**
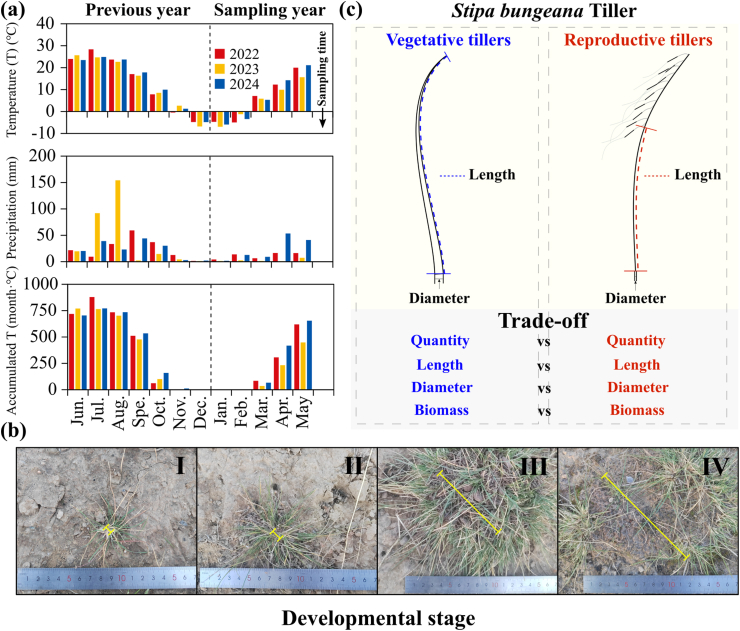
Monthly precipitation, mean monthly temperature, and accumulated temperature in the study area from 2022 to 2024 (a); photographs of *Stipa bungeana* at four developmental stages (I–IV) based on basal diameter (indicated by yellow line) (b); schematic diagrams of various measurement indices for *Stipa bungeana* (b, c). Basal diameter of *Stipa bungeana* is defined as the span formed by continuously distributed visible vegetative tillers. Tiller number is the visible surviving vegetative tillers and reproductive tillers produced in the current year per plant, respectively. Tiller length is measured from the base to the tip. Tiller diameter is measured at the middle of the tiller. Tiller biomass is expressed on a per-plant basis.

*Stipa bungeana*, a perennial, densely tufted grass species of the Poaceae family, is a dominant species of typical steppe plant communities (Fig. 1b). During individual development, plants progress through distinct biological development states, each characterized by a specific combination of quantitative and qualitative traits, such as the proportion of living to dead structures and the balance between actively growing and fully developed tissues [24]. Similar to most densely tufted species in the Poaceae family, *S. bungeana* undergoes clonal reproduction via vegetative tillers and sexual reproduction via reproductive tillers that bear seeds. As *S. bungeana* continuously produces new vegetative tillers at the base, thereby increasing its basal diameter, we classified individual plants into different developmental stages based on basal diameter (Fig. 1b). *S. bungeana* generally resume growth in early spring, from late March to early April, with seed maturation occurring by early June. Aboveground biomass remains viable until September, followed by senescence and death between October and February of the following year, while the basal tillers survive throughout this period.

**Fig. 2:**
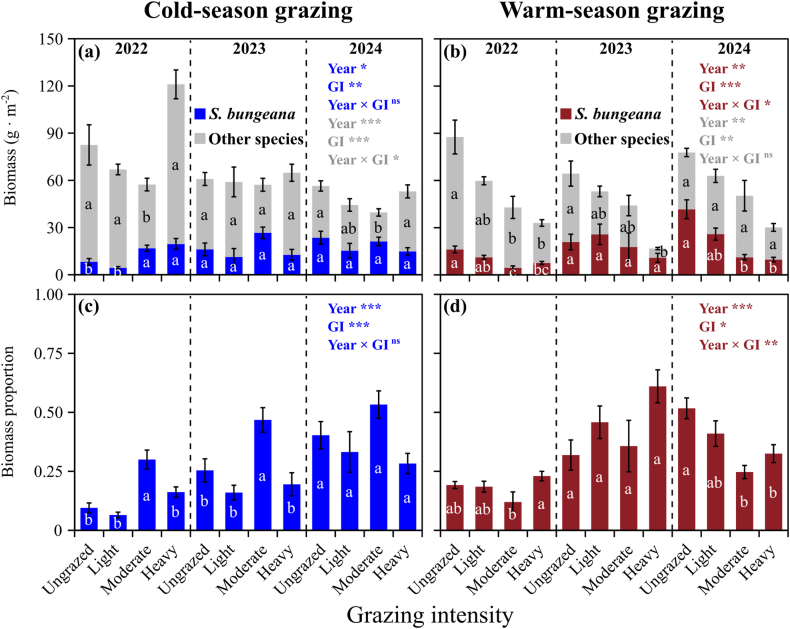
Biomass of *Stipa bungeana* (a, b) and its proportion in the plant community (c, d) in response to grazing intensity (GI) and year. Lowercase letters indicate statistically significant differences (*P* < 0.05) across grazing gradients within a year, based on Tukey's HSD test. Results of the two-way ANOVA are indicated as follows: ∗∗∗*P* < 0.001, ∗∗*P* < 0.01, ∗*P* < 0.05, ns (not significant) *P* > 0.05.

*S. bungeana* is a valuable forage grass highly preferred by goats and sheep. It is tolerant of trampling and possesses significant productive and pastoral value. Simultaneously, *S. bungeana* contributes substantially to the productivity of plant communities in typical steppes of China. The biomass of *S. bungeana* remained relatively stable and maintained a high proportional contribution under different long-term cold-season grazing intensities (Fig. 2a, b). Even under long-term warm-season heavy grazing conditions, *S. bungeana* still contributed 38.8% of the plant community's biomass (Fig. 2d). These results indicate that as a dominant species in the grassland plant community, *S. bungeana* plays a crucial role in stabilizing and sustaining grassland productivity under the interactive effects of varying climatic conditions and grazing intensities.

### 2.3 Climate data

Climate data used in this study were sourced from the historical daily database provided by Ventusky (https://www.ventusky.com/). As field sampling was conducted in early June each year, we compiled climate data for a 12-month period spanning from June 1 of the previous year to May 31 of the sampling year to represent the annual climate conditions preceding sampling (Fig. 1a). Based on these daily records, we calculated three key climatic variables: 1) annual precipitation (precipitation) was obtained by summing all daily precipitation; 2) mean annual temperature (temperature) was derived by first averaging temperatures recorded at 02:00, 08:00, 14:00, and 20:00 local time for each day, and then averaging these daily mean values across the period; and 3) annual accumulated temperature (>10 °C) was calculated by summing all daily mean temperatures that exceeded the 10 °C threshold.

### 2.4 Grazing experimental design

**Fig. 3:**
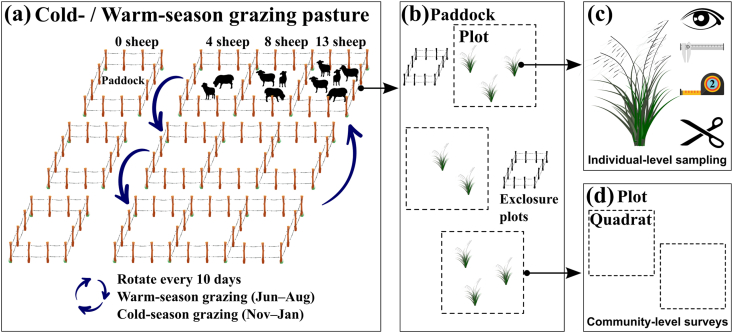
Schematic diagrams of grazing experiment management, observation, and sampling. Diagram of seasonal rotational grazing management (a); diagram of sampling plot layout (b); diagram of individual-level observation and sampling (c); diagram of community-level quadrat layout (d).

The grazing management experimental platform was established in 2001 to investigate the effects of seasonal rotational grazing under varying intensities (Fig. 3a). Male Tan sheep (*Ocis aries*), averaging 30 kg in body weight, were used for grazing and were divided into three fixed groups of 4, 8, and 13 sheep, representing light (LG), moderate (MG) and heavy (HG) grazing intensities, respectively.

The experiment comprised 21 independent paddocks, each covering an area of 0.5 ha (50 m × 100 m). Among these, 18 paddocks were assigned to grazing treatments: nine for warm-season grazing (from June to August each year) and nine for cold-season grazing (from November to January the following year). In both seasons, grazing was conducted in three rotational cycles per month, with each cycle lasting 10 days. All paddocks maintained fixed grazing intensities from experimental platform established. Each grazing intensity level was replicated across three separate paddocks per season. The remaining three paddocks served as undisturbed controls. Each paddock constituted a true experimental unit.

In addition, every paddock contained two exclosure plots (ungrazed, 3 m × 3 m each), which were established to provide ungrazed reference plants from the same local environment as those subject to grazing.

### 2.5 Sampling and measurement

This study aimed to evaluate the effects of different grazing management practices on *S. bungeana*, focusing on traits of vegetative and reproductive tillers, trait trade-offs and associations, resource allocation patterns, and individual biomass accumulation mechanisms. Individual-level sampling was conducted in early June of 2022, 2023, and 2024, coinciding with the seed maturity stage. To eliminate potential confounding effects resulting from rotational grazing cycles within any single month over the entire grazing period, three independent and synchronized paddocks under varying grazing intensities were selected for sampling during both cold and warm seasons. Within each paddock, three 20 m × 20 m plots were established, from which individual *S. bungeana* plants bearing reproductive tillers were randomly selected, with a minimum inter-plant spacing of 1 m maintained. In addition, undisturbed *S. bungeana* samples were collected from all exclosure plots located within the selected paddocks (Fig. 3b, c).

To further assess and clarify the role of *S. bungeana* at the plant community level in the study area, community-level vegetation surveys were carried out in mid-August (the peak growing season) of 2022, 2023, and 2024, within the same paddocks where individual-level sampling had been performed in early June (Fig. 3d). Specifically, three 5 m × 5 m plots were randomly established in each paddock, and two 1 m × 1 m quadrats were then randomly placed within each plot. In each non-grazed exclosure, two randomly located 1 m × 1 m quadrats were designated as controls. All aboveground plant material within each quadrat was cut at ground level using scissors, sorted by species, placed into paper bags, and transported to the laboratory. The fresh samples were oven-dried at 105 °C for 10 min, then dried at 65 °C to constant weight to determine biomass.

The measured tillering traits were selected for their specific ecological and functional significance. The number of tillers represents the plant's investment in clonal expansion (vegetative tillers) or reproductive effort (reproductive tillers). Tiller length is associated with light capture ability and competitive stature within the canopy. Tiller diameter relates to mechanical strength, resource storage capacity, and hydraulic conductance. The total biomass of vegetative or reproductive tillers reflects the overall resource allocation to these functions at the individual plant level. Specifically, we first counted the number of vegetative and reproductive tillers on each plant. We then used a steel tape measure to measure the plant's basal diameter, the length along the tiller, and the diameter of the tiller with a digital caliper. We then carefully cut the bottoms of the vegetative and reproductive tillers with scissors and placed them in separate paper bags. All plant samples were transported to the laboratory and dried at 65 °C until a constant weight was achieved for biomass measurement.

### 2.6 Calculations and statistical analysis

#### 2.6.1 Experimental factors and response variables

We evaluated the effects of grazing season (warm-season, cold-season), grazing intensity (light: 4 sheep, moderate: 8 sheep, heavy: 13 sheep, ungrazed), climate factors (annual temperature, precipitation, accumulated temperature), and individual developmental stage (as a continuous variable represented by basal diameter) on a suite of tillering traits (quantity, length, diameter, biomass) and reproductive investment.

To elucidate the dominant status of *S. bungeana* in the plant community, we monitored its population biomass and its percentage share of the total community biomass. This investigation was carried out in grazed pastures with different intensity levels during the cold and warm seasons throughout a three-year monitoring period (2022-2024). The data were subsequently subjected to analysis of variance (ANOVA) to determine the statistical significance of the variations (Fig. 2). These analyses were performed using JASP software (Jeffreys's Amazing Statistics Program, Version 0.18.3; JASP Team, 2023, available at https://jasp-stats.org/).

#### 2.6.2 Analysis of trait comparisons

We used general linear mixed-effects models from the ‘lme4’ R package to analyze the effects of sampling year and grazing intensity on different traits of vegetative and reproductive tillers of *S. bungeana* (Table S1 [25]). To isolate season-specific mechanisms, we stratified the analyses by grazing season, fitting separate models for the cold-season and warm-season datasets. In each season-specific model, sampling year (Year), grazing intensity (GI), and their interaction (Year: GI) were treated as fixed effects. Plot was included as a random intercept factor, with the model structure specified as Y ∼ Year + GI + Year × GI + (1 | Plot).

**Fig. 4:**
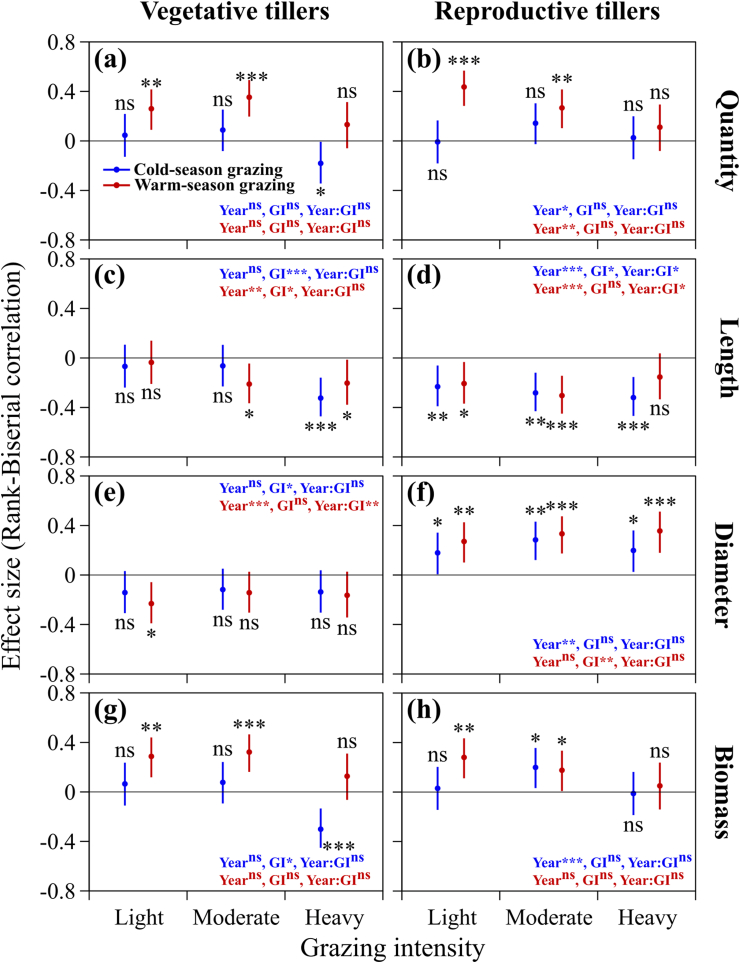
Effects of grazing intensity on vegetative and reproductive tiller traits of *Stipa bungeana* in cold- and warm-season grazing pastures (2022–2024), relative to ungrazed exclosures. Panels show tiller quantity (a, b), length (c, d), diameter (e, f), and biomass (g, h). Effect sizes are shown as rank-biserial correlations with 95% confidence intervals. Positive values indicate an increase in the trait under grazing, and negative values indicate a decrease. Significance levels: ∗*P* < 0.05, ∗∗*P* < 0.01, ∗∗∗*P* < 0.001; ns, not significant. Blue and red letters indicate significance levels for the effects of sampling year (Year), grazing intensity (GI), and their interaction (Year:GI) on different vegetative and reproductive tiller traits in cold-season and warm-season pastures, respectively, based on general linear mixed-effect models. ∗*P* < 0.05, ∗∗*P* < 0.01, ∗∗∗*P* < 0.001; ^ns^*P* ≥ 0.05.

To further compare the degree of change in tiller traits between different grazing treatments and the non-grazing control, we calculated non-parametric effect sizes (rank biserial correlation) from Mann-Whitney tests for visualization (Fig. 4). Crucially, the linear mixed-effects framework serves as our primary basis for formal statistical inference across the experimental design, whereas the Mann-Whitney results are intended solely as descriptive summaries of effect-size comparisons relative to the ungrazed control. These analyses were performed using JASP software.

#### 2.6.3 Trade-off analyses

We calculated the root-mean-square error (RMSE) among four vegetative and reproductive tiller traits (number, length, diameter, biomass) of *Stipa bungeana* to evaluate trait-dispersion-based allocation shifts [26]. Prior to calculating RMSE, each of traits was standardised to a [0, 1] scale across all individual plants in the dataset using the observed minimum and maximum values:

(1) $${Traits}_{std}=\frac{{Traits}_{obs}\;-\;{Traits}_{\min}}{{Traits}_{\max}\;-\;{Traits}_{\min}}$$

where *Traits*_*obs*_ is the observed raw value of a given trait for an individual plant; *Traits*_*min*_ and *Traits*_*max*_ are the minimum and maximum observed values of that same trait across all individuals in the dataset.

The RMSE for each trait was then calculated within each paddock across all *n* individual plants sampled:

(2) $$RMSE=\sqrt{\frac{1}{\left(n\;-\;1\right)}\times \sum_{i=1}^{n}{\left({Traits}_{i}\;-\;\bar{Traits}\right)}^{2}}$$

Where *Traits*_*i*_ is the standardised value (from Equation (1)) of the given trait for the *i*-th individual plant (*i* = 1, 2, …, *n*); $\bar{T\text{raits}}$ is the mean standardised value of that same trait across all *n* individuals within the paddock, representing the within-paddock benchmark; and *n* is the number of individual plants sampled per paddock. The RMSE, derived from the deviation of individual observations from this benchmark, quantifies the degree of variation in that trait across different grazing treatments.

**Fig. 5:**
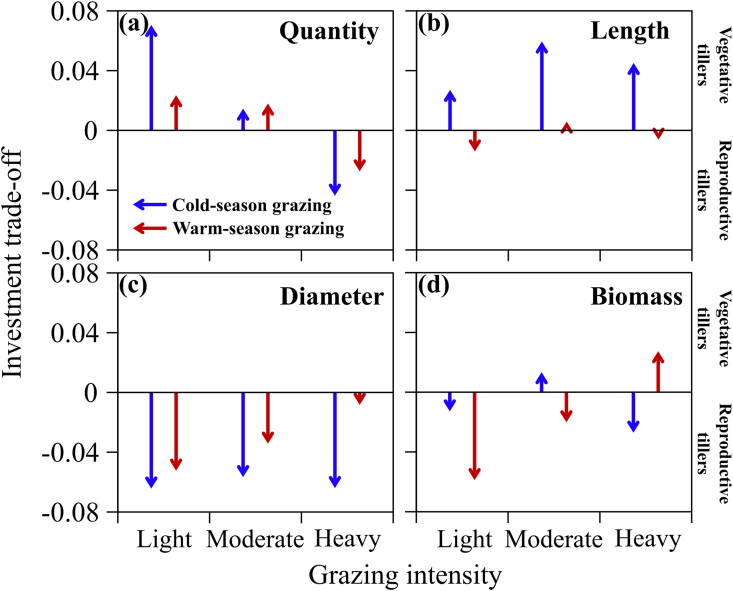
Effects of grazing season and intensity on trait trade-offs between vegetative and reproductive tillers in *Stipa bungeana*. Arrows represent the allocation balance for tiller quantity (a), length (b), diameter (c), and biomass (d). Arrow length indicates the strength of allocation preference, with direction (positive/negative) denoting the shift toward vegetative or reproductive investment. Arrow values were calculated as the difference in trade-off values between grazed and ungrazed conditions.

Further, we assigned the RMSE values of different traits for vegetative and reproductive tillers under various grazing treatments as the x and y coordinates in a two-dimensional plane, respectively, and calculated the perpendicular distance from each point (x, y) to the 1:1 dashed line. This distance represents the allocation imbalance between vegetative and reproductive tillers for that trait under the given treatment. Because RMSE mathematically characterizes trait dispersion and variation away from a paddock benchmark rather than a direct physiological mechanism, this index is interpreted as a reflection of allocation imbalance driven by trait-dispersion shifts. Finally, we computed the difference in allocation imbalance between each grazing treatment and the non-grazing treatment for vegetative and reproductive tillers, and plotted these differences (Fig. 5). A positive value indicates that the grazing treatment shifts the trade-off toward vegetative tillers, whereas a negative value indicates a shift toward reproductive tillers.

#### 2.6.4 Network analyses

**Fig. 6:**
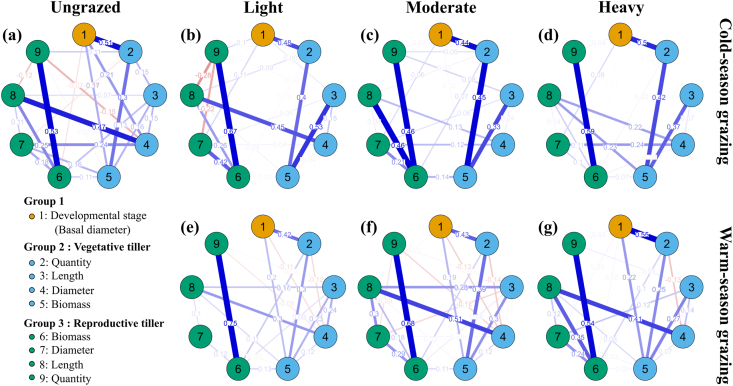
Network analysis of relationships among developmental stage (basal diameter), vegetative and reproductive tiller traits, and tillering allocation in *Stipa bungeana* under different grazing intensities in ungrazed exclosures (a), cold-season (b–d), and warm-season grazing pastures (e–g). Edge width and label indicate partial correlation weight; blue edges = positive associations; red edges = negative associations. The maximum edge weight is indicated in each panel.

We used a network graph to visually represent the correlation between the vegetative and reproductive tillers traits of *S. bungeana* under various grazing management measures (Fig. 6). First, we transformed the data logarithmically to meet the requirements of normal distribution and network analysis. Then we estimated it with the EBICglasso method, and the network graph was created and outputted with JASP software.

To assess the robustness of the estimated networks, we performed non-parametric bootstrapping (1000 iterations) for each of the seven treatment networks using JASP software. For each bootstrap replicate, the EBICglasso procedure was re-applied to a sample drawn with replacement from the original dataset, yielding a distribution of edge weights for every pair of nodes. Bootstrapped 95% confidence intervals (CIs) surrounding each edge weight were used to evaluate estimation accuracy: edges whose CIs excluded zero were considered reliably estimated and were the primary basis for mechanistic interpretations. Additionally, centrality stability was assessed via case-dropping subset bootstrapping, in which the correlation stability heatmaps visualize the consistency of node centrality rankings (Strength, Betweenness, and Closeness) as increasing proportions of cases are progressively removed. Nodes maintaining stable ranking positions across subsets indicate robustly estimated centrality. In accordance with established recommendations [27], Strength and Expected Influence, which generally exhibit higher stability than Betweenness and Closeness, were treated as the primary centrality metrics for comparative interpretation. All bootstrap and stability results are provided in Supplementary Table S2–S4 and Fig. S1–S3.

#### 2.6.5 Random forest models

**Fig. 7:**
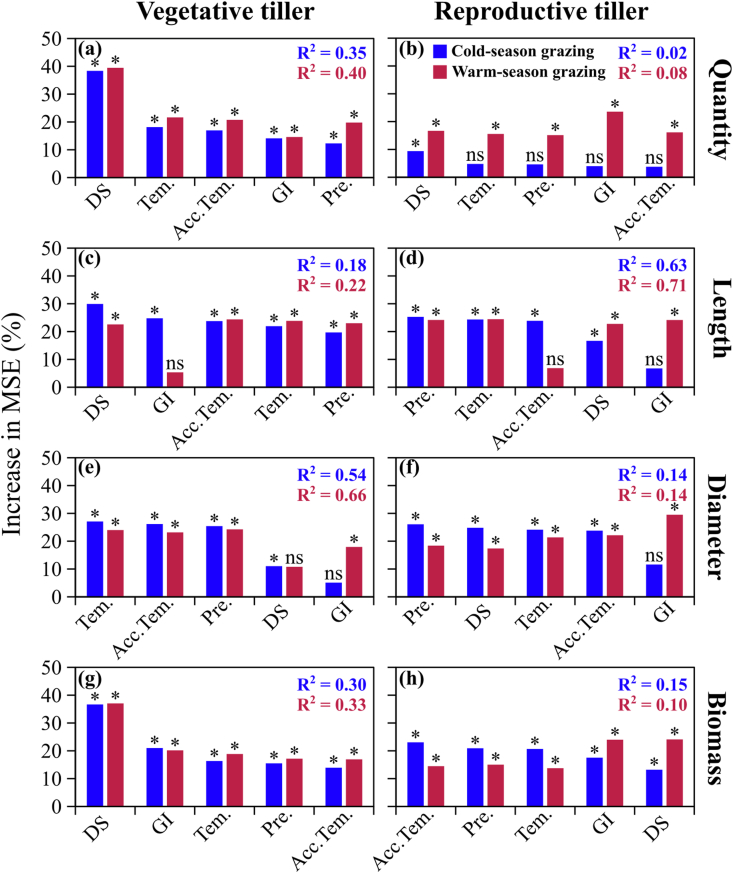
Relative contributions of various factors to vegetative and reproductive tiller traits in *Stipa bungeana*, as estimated by random forest models. Panels show results for tiller quantity (a, b), length (c, d), diameter (e, f), and biomass (g, h) in cold- and warm-season grazing pastures. Asterisks (∗) denote significance at *P* < 0.05; "ns" indicates non-significant effects. R^2^ in the figure represents the model's variance explained. Abbreviations: DS, developmental stage (basal diameter); Tem., temperature; Pre., precipitation; Acc.Tem., accumulated temperature; GI, grazing intensity.

Random forest models were implemented using the 'randomForest' R package [28] to determine the relative importance of five predictors, grazing intensity, accumulated temperature, annual temperature, annual precipitation, and individual size (basal diameter), for each tillering trait response variable (Fig. 7). Each model was constructed with 1000 trees and the number of variables sampled at each node split was set to the default value for regression tasks. Variable importance was quantified as the percentage increase in mean squared error (%IncMSE) upon permutation of each predictor out-of-bag. Significance of variable importance was assessed using a permutation test (500 permutations) implemented in the ‘rfPermute’ package, in which the observed %IncMSE was compared against a null distribution constructed by randomly shuffling the response variable. Models were fitted separately for the cold-season and warm-season grazing pastures datasets.

#### 2.6.6 Path models

**Fig. 8:**
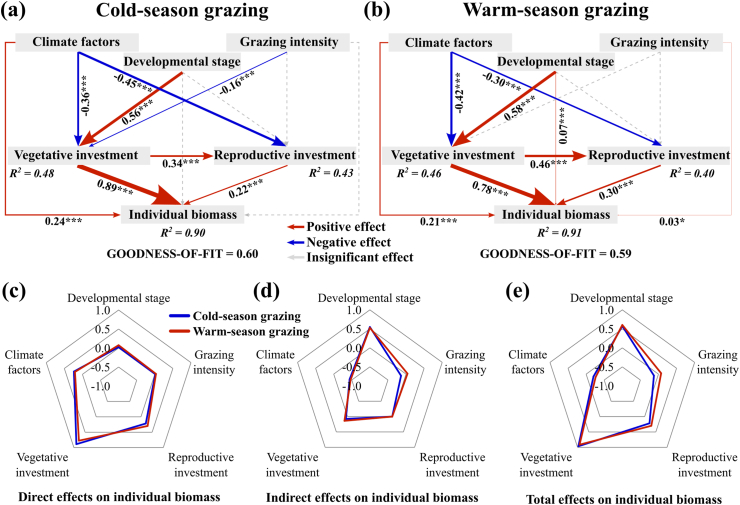
Partial least squares path modeling (PLSPM) illustrating the pathways through which climate factors, grazing intensity, and developmental stage (basal diameter) influence individual biomass of *Stipa bungeana* in cold- and warm-season grazing pastures via vegetative and reproductive tillers (a, b). Climate factors comprised annual temperature, annual precipitation, and accumulated temperature; vegetative and reproductive investment included tiller quantity, length, diameter, and biomass. Red arrows denote positive effects, blue arrows negative effects, and gray arrows non-significant effects; numbers adjacent to arrows represent direct effect sizes and significance levels (∗∗∗*P* < 0.001, ∗∗*P* < 0.01, ∗*P* < 0.05). The radar chart displays the direct (c), indirect (d), and total (e) effects of each variable on individual biomass in cold- and warm-season grazing pastures, respectively.

Partial least squares path models (PLSPM) were constructed separately for cold-season and warm-season pastures using the ‘plspm’ R package [29] to examine the causal pathways through which climate, developmental stage (basal diameter), and grazing intensity influence individual biomass of *S. bungeana* via vegetative and reproductive investment (Fig. 8). Five latent variables were defined: (1) Climate, with annual temperature, annual precipitation, and accumulated temperature as reflective indicators; (2) Vegetative investment, with vegetative tiller number, length, diameter, and biomass as reflective indicators; (3) Reproductive investment, with reproductive tiller number, length, diameter, and biomass as reflective indicators; (4) Developmental stage and (5) Individual biomass, each specified as single-indicator constructs. Overall model fit was evaluated using the Goodness-of-Fit (GoF) index, computed as the geometric mean of the average communality and the average R^2^ across endogenous latent variables; GoF >0.36 was adopted as the threshold for a globally valid model. R^2^ values for each endogenous variable are reported within Fig. 8.

#### 2.6.7 Prediction equations

To examine predictive relationships among predicted vegetative and reproductive investment intensity, as well as the formation of individual biomass, we developed a prediction equation using the multiple linear stepwise regression method, which was run in JASP software (Table 1). To assess model generalizability, each stepwise regression equation was subjected to 5-fold cross-validation using the caret package [30] in R. The dataset for each pasture type was randomly partitioned into five equal subsets; each subset served once as the validation set while the remaining four were used for training. The cross-validated R^2^ (R^2^cv) was computed as the mean R^2^ across the five held-out folds. Given the hierarchical structure of our data (nested within years and plots), this random cross-validation partitions individuals such that identical environmental blocks exist across training and testing folds. Therefore, the cross-validated R^2^ (R^2^cv) is interpreted strictly as an indicator of internal model stability and reproducibility within our experimental framework, rather than an assertion of broader geographic or temporal predictive generalizability. Models showing R^2^cv within 0.05 of Adj-R^2^ were considered stable.

**Table 1:** Prediction equations for vegetative and reproductive investment and individual biomass in cold- and warm-season pastures.

Pasture	Model	Adj-R^2^	R^2^cv	*P*
Ungrazed	IndBiomass = VegTillerN × 0.047 + VegTillerL × 0.056 + RepTillerN × 0.123 + RepTillerD × 0.972 - 1.306	0.63	0.628	<0.001
Cold-season	IndBiomass = VegTillerN × 0.081 + VegTillerL × 0.114 + RepTillerN × 0.087 + RepTillerL × 0.009 - 1.515	0.91	0.913	<0.001
Warm-season	IndBiomass = BasalDiam × 0.057 + VegTillerN × 0.053 + VegTillerL × 0.079 + VegTillerD × 1.421 + RepTillerN × 0.082 + RepTillerL × 0.017 - 1.783	0.85	0.861	<0.001
All	IndBiomass = BasalDiam × 0.041 + VegTillerN × 0.058 + VegTillerL × 0.096 + RepTillerN × 0.083 + RepTillerL × 0.015 - 1.357	0.83	0.822	<0.001

Note: The prediction equations were developed using linear stepwise regression, with model selection based on maximizing the adjusted R^2^, minimizing the root mean squared error (RMSE), and minimizing the number of parameters. Cross-validated R^2^ (R^2^cv) was estimated by 5-fold cross-validation (repeated with seed = 42 for reproducibility) using the caret package in R. Models in which R^2^cv differed from Adj-R^2^ by less than 0.05 were considered generalizable. VegTiller = vegetative tiller, RepTiller = reproductive tiller; N = number, L = length, D = diameter.

## 3 Results

### 3.1 Effects of grazing on vegetative and reproductive tiller traits

Warm-season grazing increased the number of both vegetative and reproductive tillers to varying degrees, while cold-season grazing showed no significant effect (Fig. 4a and b). Heavy grazing significantly reduced the length of vegetative tillers, and grazing in both seasons shortened reproductive tillers (Fig. 4c and d). In contrast, grazing did not significantly influence the diameter of vegetative tillers but led to a notable increase in the diameter of reproductive tillers (Fig. 4e and f). With regard to biomass, light to moderate warm-season grazing enhanced both vegetative and reproductive tiller biomass, whereas heavy cold-season grazing reduced vegetative tiller biomass, and moderate warm-season grazing increased reproductive tiller biomass (Fig. 4g and h).

### 3.2 Trait-dispersion-based allocation shifts between tiller types

Analysis of allocation imbalances between vegetative and reproductive tiller traits in *S. bungeana* revealed that light to moderate grazing favored greater allocation to vegetative tiller number, while heavy grazing shifted investment away from it (Fig. 5a). Cold-season grazing increased allocation to vegetative tiller length, whereas warm-season grazing had no significant influence on the length allocation pattern between the two tiller types (Fig. 5b). Grazing generally led to thicker reproductive tillers (Fig. 5c). Under warm-season grazing, light to moderate intensity promoted biomass allocation to reproductive tillers, in contrast to heavy grazing which suppressed it. Meanwhile, moderate cold-season grazing enhanced investment in vegetative tiller biomass (Fig. 5d).

### 3.3 Trait coordination and network rewiring under grazing

Before interpreting specific network features, we verified the robustness of all seven estimated networks via non-parametric bootstrapping (1000 iterations; Fig. S1–S3, Table S2–S4). Two edges showed consistently strong and reliably estimated weights across all treatments: the RepTillerN–RepTillerB edge (weights: 0.456–0.749; bootstrap CIs excluding zero in all networks) and the BasalDiam–VegTillerN edge (weights: 0.423–0.550), confirming that reproductive tiller biomass and vegetative tiller number are robustly linked to their respective anchors regardless of grazing regime (Table S4). Among centrality indices, Strength and Expected Influence were stable across networks, with RepTillerB consistently ranking as the most strongly connected node in six of seven treatments (Table S3). Betweenness and Closeness showed greater variability and are treated as exploratory. Network-level connectivity declined progressively under cold-season grazing (NG: 23 edges; CHG: 18 edges), while warm-season moderate grazing produced the densest network (24 edges), exceeding the ungrazed reference (Table S2).

In cold-season pastures, grazing strengthened positive correlations among vegetative tiller number, length, and biomass, while eliminating the positive links between vegetative tiller diameter, biomass, and basal diameter that were reliably present in the ungrazed network (CIs excluding zero in NG; absent in cold-season grazed networks; Table S4). This pattern indicates grazing-induced decoupling of plant size from vegetative structural traits. Light grazing intensified the negative correlation between reproductive tiller length and number, but moderate to heavy grazing removed this relationship; moderate grazing additionally enhanced the positive correlation between reproductive tiller length and biomass (Fig. 6a, b, c, d). Grazing also eliminated the negative link between vegetative tiller diameter and reproductive tiller number, and weakened the coupling between vegetative tiller diameter and reproductive tiller length.

In warm-season pastures, light to moderate grazing weakened the positive relationship between basal diameter and vegetative tiller number, and reversed the positive basal diameter–vegetative tiller diameter association to a negative one, a sign reversal supported by bootstrap CIs (Table S4). Grazing removed the positive correlation between vegetative tiller number and length, while establishing a new positive correlation between vegetative tiller diameter and biomass (Fig. 6a, e, f, g). Additionally, grazing reduced the coupling between vegetative tiller diameter and reproductive tiller length, and between vegetative tiller biomass and reproductive tiller diameter, suggesting a disturbance-driven decoupling of vegetative and reproductive modules.

Together, these results show that grazing does not merely suppress or promote individual traits but systematically rewires their coordination, with the direction of rewiring contingent on grazing season: cold-season grazing simplifies the network by severing size-dependent linkages, while warm-season grazing reshapes integration by establishing new morphological associations.

### 3.4 Key drivers of tiller trait variation

Basal diameter exerted the strongest influence on the number of vegetative tillers in *S. bungeana* (Fig. 7a), while in warm-season grazing pastures, grazing intensity was the primary factor affecting the number of reproductive tillers (Fig. 7b). Both climate conditions and basal diameter significantly affected the length of vegetative and reproductive tillers. Specifically, cold-season grazing notably influenced vegetative tiller length, whereas warm-season grazing had a stronger effect on reproductive tiller length (Fig. 7c and d). Climate conditions also played a significant role in determining tiller diameter for both vegetative and reproductive tillers (Fig. 7e and f), though in warm-season pastures, grazing intensity had the greatest impact on reproductive tiller diameter (Fig. 7f). Regarding biomass, basal diameter was the dominant factor for vegetative tiller biomass (Fig. 7g). For reproductive tiller biomass, accumulated temperature was the most influential factor in cold-season grazing pastures, whereas in the warm-season grazing pasture, grazing intensity and basal diameter had the greatest impact on the biomass of reproductive tillers (Fig. 7h).

### 3.5 Pathways governing individual biomass accumulation

Climate exerted a significant negative effect on both vegetative and reproductive investment, whereas basal diameter showed a significant positive effect on vegetative investment (Fig. 8a and b). Vegetative investment positively influenced reproductive investment, and both investment pathways contributed to individual biomass accumulation. Furthermore, climate factors exhibited a direct positive effect on individual biomass (Fig. 8a and b). Among all factors, vegetative investment had the strongest direct effect on individual biomass, while basal diameter exerted the greatest indirect influence (Fig. 8c and d). Overall, both vegetative investment and basal diameter played significant roles in determining individual biomass (Fig. 8e).

### 3.6 Predictive models for plant investment and biomass

Across all pasture types, the quantity and length of both vegetative and reproductive tillers significantly influenced individual biomass. Basal diameter significantly affected individual biomass in warm-season pastures, but showed no significant effect in enclosed or cold-season pastures. Overall, individual biomass was primarily governed by basal diameter, together with the quantity and length of vegetative and reproductive tillers (Table 1).

## 4 Discussion

### 4.1 Grazing-driven modifications in tiller morphology

Warm-season grazing increased the number of both vegetative and reproductive tillers, whereas cold-season grazing showed no significant effect (Fig. 4a and b). This pattern aligns with our first hypothesis that warm-season grazing exerts stronger stimulatory effects on tiller production. The outcome may be attributed to two main factors. First, continuous foraging during the active growth period disrupts apical dominance, thereby stimulating compensatory regeneration, particularly of vegetative tillers [31]. Second, during the cold season, plants are largely dormant, and tillering-related physiological processes exhibit limited responsiveness to grazing stimuli [32].

Grazing during both seasons reduced the length but increased the diameter of reproductive tillers (Fig. 4d and f). This structural adjustment supports the notion of grazing-induced phenotypic plasticity. Shorter, thicker reproductive tillers likely confer greater lodging resistance, which may be adaptive in disturbance-prone environments [33].

Grazing had a minimal effect on the diameter but significantly reduced the length of vegetative tillers under heavy grazing (Fig. 4c and e). The maintenance of tiller diameter may help preserve photosynthetic capacity, while reduced height can enhance spatial avoidance, collectively minimizing grazing intake [17]. Moderate grazing intensity increased reproductive tiller biomass in both seasons (Fig. 4g and h). This may reflect a strategic shift where moderate disturbance stimulates compensatory growth, freeing up resources for reproductive development [34], and potentially promoting higher seed investment for dispersal away from grazed areas [35,36].

Overall, these morphological changes support our first hypothesis. Warm-season grazing more effectively stimulated tiller production, while the reduction in reproductive tiller length and increase in diameter represent clear plastic responses to grazing pressure.

### 4.2 Shifts in allocation strategies between reproduction modes

Light to moderate grazing favored allocation to vegetative tiller number, whereas heavy grazing shifted investment towards reproductive tillers (Fig. 5a). This finding aligns with the classic resource allocation theory under disturbance. Under lower pressure, enhancing clonal propagation secures spatial occupancy [18]. Under heavy grazing, where persistent defoliation threatens survival, increasing investment in sexual reproduction may become a strategy to ensure population persistence through dispersal [37,38].

Cold-season grazing increased allocation to vegetative tiller length, while warm-season grazing had no significant effect on length-related investment (Fig. 5b). This pattern may be attributed to livestock excreta deposited during winter, combined with low temperatures that slow microbial activity, thereby preserving nutrient availability. These retained nutrients become accessible for plant uptake during spring green-up [39]. In contrast, warm-season grazing occurs from June to August, after *S. bungeana* has largely completed its reproductive cycle. Grazing and associated excretion during this period may instead accelerate growth and nutrient depletion through compensatory responses, thereby increasing the influence of climate on subsequent tiller length investment [34,40].

Grazing consistently increased the relative diameter of reproductive compared to vegetative tillers (Fig. 5c). This suggests a prioritized strengthening of reproductive structures, possibly to protect developing seeds and ensure reproductive success under grazing pressure, while maintaining the essential photosynthetic function of vegetative tillers with less structural modification [41,42].

Our second hypothesis proposed that cold-season grazing simplifies trait correlation networks. The network analyses (Fig. 6) confirm this by showing a reduction in integrative links among traits like basal diameter, tiller diameter, and biomass under cold-season grazing. Conversely, warm-season grazing maintained or even established new correlations (e.g., between tiller diameter and biomass). This indicates that grazing during dormancy weakens internal phenotypic coordination, potentially making plants more susceptible to external climatic drivers, whereas grazing during the growth period acts as a force that reshapes but does not necessarily dismantle functional trait integration.

### 4.3 Decoupling and reorganization of trait relationships

Cold-season grazing strengthened the positive correlations among the quantity, length, and biomass of vegetative tillers (Fig. 6a, b, c, d). This integration of traits likely resulted from reduced canopy cover after grazing, which improved light availability at the plant base and alleviated light competition. These conditions collectively promoted the synchronous increase in the number, elongation, and biomass accumulation of vegetative tillers during the subsequent greening period [22]. Cold-season grazing was associated with the disappearance of the positive correlations between vegetative tiller diameter, biomass, and basal diameter observed in the ungrazed network; bootstrap confidence intervals indicate that these edges were present and reliably estimated in the ungrazed network (CIs excluding zero) but absent in the cold-season grazed networks, supporting the interpretation of grazing-induced network simplification (Fig. 6a, b, c, d). This decoupling can be attributed to the removal of a large proportion of senesced tillers during cold-season grazing, which facilitated the emergence of new, smaller-diameter tillers in the following growing season, thereby disrupting the positive relationship between basal diameter and biomass. Moderate grazing enhanced the positive correlation between reproductive tiller length and biomass (Fig. 6a, b, c, d), possibly because such grazing intensity stimulated compensatory growth, leading to greater dry matter accumulation in reproductive tillers [43].

Under warm-season grazing at light to moderate intensities, the positive relationship between basal diameter and the number of vegetative tillers was weakened (Fig. 6a, c, f). This likely occurred because grazing disturbance redirected resources toward compensatory growth rather than toward the simultaneous expansion of basal area and production of new tillers [6]. Warm-season grazing was associated with the absence of the positive correlation between vegetative tiller quantity and length seen in ungrazed conditions, while a positive correlation between tiller diameter and biomass appeared in grazed networks (Fig. 6a, e, f, g); the reliability of these contrasts is supported by bootstrap confidence intervals (Fig. S1). These patterns suggest a morphological adjustment under grazing, though the causal directionality of this network rewiring remains to be confirmed by experimental manipulation. Specifically, plants appeared to reduce tiller length to improve spatial avoidance while increasing tiller diameter to maintain photosynthetic capacity and nutrient supply [17]. Grazing also reduced the positive correlations between vegetative tiller diameter and reproductive tiller length, as well as between vegetative tiller biomass and reproductive tiller diameter (Fig. 6a, e, f, g). This decoupling suggests that grazing-induced loss of vegetative tillers prompted plants to prioritize the regeneration of photosynthetic structures over investment in reproductive development [6].

These changes in trait networks support the view that grazing does not merely suppress or promote traits in isolation but rewires their relationships, and the nature of this rewiring is contingent on the grazing season, consistent with our second hypothesis.

### 4.4 Integrated mechanisms of biomass regulation under grazing

The random forest models (Fig. 7) provided direct support for our second hypothesis by quantifying the relative importance of key drivers. They showed that individual developmental stage (basal diameter) was the dominant predictor for vegetative tiller number and biomass. In contrast, reproductive tiller traits, particularly their number and diameter in warm-season pastures, were primarily governed by grazing intensity and climatic factors, especially accumulated temperature. This clear separation in primary drivers establishes a fundamental “control asymmetry”: vegetative growth capacity is intrinsically set by plant size, whereas reproductive allocation is more plastic and responsive to external pressures [44,45].

This “control asymmetry” is not merely a statistical observation; it provides the mechanistic basis for the restructuring of phenotypic integration, thereby addressing our third hypothesis. When vegetative and reproductive modules are driven by different factors (internal state vs. external environment), their coordinated expression is inevitably altered. The network analyses (Fig. 6) visualized this decoupling and rewiring. For instance, the weakened or eliminated correlations between basal diameter and reproductive tiller traits under grazing can be interpreted as a consequence of this control separation. The external driver (grazing) directly modulates reproductive investment, bypassing or disrupting its typical linkage with the plant's developmental stage. Similarly, the strengthening of correlations among vegetative tiller traits under cold-season grazing suggests a synchronized response to a common, overriding stimulus (e.g., improved light availability post-grazing), which temporarily homogenizes their variation [22].

Path modeling (Fig. 8) integrated these relationships into a causal framework, showing how developmental stage ultimately governs biomass. It confirmed that basal diameter exerts a strong positive direct effect on vegetative investment. Crucially, it also revealed a significant indirect effect of developmental stage on both reproductive investment and final individual biomass, mediated entirely through this vegetative pathway. This underscores that larger plants achieve greater reproductive output and biomass principally by sustaining a larger photosynthetic apparatus. The direct negative effect of climate on investment may reflect a conservative strategy under semi-arid conditions, while its direct positive effect on final biomass likely operates through resource availability (water, nutrients) unrelated to the measured tiller traits [21].

Finally, the predictive models (Table 1) reinforced the paramount role of developmental stage, with basal diameter consistently emerging as a key variable alongside key tiller traits. Collectively, our results demonstrate that the individual plant's developmental stage acts as a pivotal internal filter and integrator. It sets the baseline capacity for growth, mediates the translation of grazing and climatic signals into phenotypic adjustments, and thereby determines the eventual outcome for individual biomass. This mechanistic understanding moves beyond descriptive effects of grazing, providing a validated framework to explain why and how phenotypic trait networks reorganize under disturbance.

### 4.5 Methodological implications and future perspectives

All phenotypic traits in this study were manually measured using conventional field instruments following established protocols widely adopted in plant ecology and grassland science. Although this approach yields high-resolution, ecologically validated data, it is labor-intensive, time-consuming, and may introduce temporal variability when applied at larger scales. Such temporal variation in measurement timing represents an inherent limitation of large-scale manual phenotyping efforts and may introduce some background noise into the data. Despite this limitation, the use of standard grassland ecology methods, multi-year repeated sampling, and a robust statistical framework in our study substantially mitigated the potential impact of individual measurement timing errors on the overall conclusions [46,47].

These methodological considerations also highlight the transformative potential of emerging AI- and computer vision-based phenotyping technologies. High-throughput imaging approaches, including UAV-borne RGB and multispectral imaging, 3D structured light scanning, and deep learning-based trait extraction pipelines, have demonstrated great promise for rapidly and non-destructively automating the measurement of morphological traits such as cover, height, and volume in large plant populations [48,49]. For core traits similar to those in the present study, such as basal diameter, tiller number, and tiller length, UAV-acquired imagery coupled with AI-assisted image analysis could substantially reduce measurement time per experimental campaign and potentially enable near-continuous monitoring throughout the growing season. However, direct application to densely tufted species like *S. bungeana* still faces technical challenges. Reliably distinguishing individual vegetative and reproductive tillers within overlapping canopies, as well as accurately estimating fine-scale structural traits such as tiller diameter, remains technically demanding and requires the development and validation of species-specific training datasets. Future studies that integrate these high-throughput phenotyping tools with long-term experimental platforms will greatly enhance the spatial and temporal resolution of individual-level monitoring, bridging the gap between plot-scale mechanistic insights and landscape-scale management applications.

## 5 Conclusions

This study is based on a grazing management experimental platform that has been continuously operating for over two decades, together with a three-year consecutive plant phenotyping survey, and reveals an asymmetric control structure in which plant individual developmental stage (proxied by basal diameter) predominantly governs vegetative capacity and biomass, while external drivers (climate, grazing intensity) primarily regulate reproductive investment. Furthermore, grazing season acts as a key modulator of phenotypic integration, with cold-season grazing simplifying trait correlation networks and warm-season grazing stimulating tiller production and rewiring trait relationships. These findings compel a paradigm shift from uniform grazing standards towards adaptive strategies that account for intrinsic biological heterogeneity, underscoring the value of monitoring plant individual developmental stage as a biologically meaningful indicator for precision grassland management and long-term ecosystem resilience.

## Author contributions

Y.W.Z conceived and designed the project. Y.W.Z, Z.X.G, L.L and J.Y.Z performed the experiments. Y.W.Z wrote the manuscript. Y.W.Z, L.L and F.J.H revised the manuscript. All authors discussed the results and approved the final manuscript.

## Funding

This research was funded by the 10.13039/501100012166National Key Research and Development Program (2025YFE0212000), the 10.13039/501100001809National Natural Science Foundation of China for Youth Science Fund (42407372), Innovation Platform Plan Program of Gansu Province (26JDWA001), Program for Innovative Research Team of Ministry of Education (IRT_17R50).

## Declaration of interests

The authors declare that they have no known competing financial interests or personal relationships that could have appeared to influence the work reported in this paper.

## Data Availability

The primary data associated with this study are available in the Supplementary data files.
