# Supplementary material for: Internal and external factors drive vegetative-reproductive strategies and reveal plant developmental stage as a lever for precision grassland management
Source: Plant Phenomics. 2026 Jul 8;8(3):100254. doi: 10.1016/j.plaphe.2026.100254 (PMC13400394; doi:10.1016/j.plaphe.2026.100254)
Supplement: Multimedia component 1 [file mmc1.docx]

**Supplementary Material**

***for***

**Internal and external factors drive vegetative-reproductive strategies and reveal plant developmental stage as a lever for precision grassland management**

Yu-Wen Zhang ^a^, Jie-Yan Zhou ^a^, Zhao-Xia Guo ^a^, Lan Li ^a,^*, Fu-Jiang Hou ^a,^*

^a^ China-Kazakhstan Belt and Road Joint Laboratory on Grassland Ecological Restoration, Key Laboratory of Grassland Livestock Industry Innovation, Ministry of Agriculture and Rural Affairs, Engineering Technology Research Center for Ecological Restoration and Utilization of Degraded Grassland in Northwest China, National Forestry and Grassland Administration, College of Pastoral Agriculture Science and Technology, Lanzhou University, Lanzhou 730020, China

* For correspondence. lanli@lzu.edu.cn or [cyhoufj@lzu.edu.cn](mailto:cyhoufj@lzu.edu.cn)

**This file includes:**

**Table S1-S4**

**Figure S1-S3**

**Table S1** General linear mixed-effects models were used to analyze the effects of sampling year and grazing intensity on various vegetative and reproductive tiller traits in cool-season and warm-season pastures.

| Index | Cold-season grazing | | | Warm-season grazing | | |
| --- | --- | --- | --- | --- | --- | --- |
|  | Year | GI | Year : GI | Year | GI | Year : GI |
| VegTillerN | -0.10ns | -0.09ns | 0.02ns | -0.06ns | 0.09ns | -0.01ns |
| VegTillerL | 0.04ns | -0.20*** | 0.05ns | -0.18** | -0.11* | 0.03ns |
| VegTillerD | 0.04ns | -0.13* | 0.02ns | -0.27*** | -0.05ns | -0.15** |
| VegTillerB | 0.00ns | -0.13* | 0.02ns | -0.08ns | 0.10ns | -0.06ns |
| RepTillerN | 0.12* | 0.01ns | 0.05ns | 0.19** | -0.02ns | -0.03ns |
| RepTillerL | -0.38*** | -0.11* | -0.12* | -0.35*** | -0.08ns | -0.13* |
| RepTillerD | 0.18** | 0.05ns | 0.01ns | -0.05ns | 0.19** | -0.10ns |
| RepTillerB | -0.20*** | 0.05ns | -0.05ns | -0.01ns | 0.02ns | -0.09ns |

Note: The fixed factors in the model were sampling year (Year), grazing intensity (GI), and their interaction (Year : GI), with plot as the random factor. Values are standardized regression coefficients. Asterisks indicate the significance of fixed effects (**P* < 0.05, ***P* < 0.01, ****P* < 0.001) or "ns" (*P* ≥ 0.05). VegTiller = vegetative tiller, RepTiller = reproductive tiller; N = number, L = length, D = diameter, B = biomass.

**Table S2** Network Summary Across Grazing Treatments.

| Treatment | Nodes | Non-zero Edges | Sparsity | Connectivity Trend |
| --- | --- | --- | --- | --- |
| NG | 9 | 23 / 36 | 0.36 | Reference |
| CLG | 9 | 22 / 36 | 0.39 | ↓ slightly |
| CMG | 9 | 19 / 36 | 0.47 | ↓↓ moderate |
| CHG | 9 | 18 / 36 | 0.50 | ↓↓↓ most sparse |
| WLG | 9 | 21 / 36 | 0.42 | ↓ slightly |
| WMG | 9 | 24 / 36 | 0.33 | ↑ denser than NG0 |
| WHG | 9 | 21 / 36 | 0.42 | ↓ slightly |

Note: Nodes = 9 (BasalDiam, VegTillerN, VegTillerL, VegTillerD, VegTillerB, RepTillerN, RepTillerL, RepTillerD, RepTillerB). Possible edges = 36 (complete graph). Networks estimated using the EBICglasso method (γ = 0.5) with log-transformation pre-processing. Non-parametric bootstrap: 1,000 iterations. Sparsity = proportion of possible edges set to zero by the EBICglasso regularisation. Lower sparsity = denser (more connected) network. NG = No-grazing; CLG = Cold-season light grazing; CMG = Cold-season moderate grazing; CHG = Cold-season heavy grazing; WLG = Warm-season light grazing; WMG = Warm-season moderate grazing; WHG = Warm-season heavy grazing; VegTiller = vegetative tiller, RepTiller = reproductive tiller; N = number, L = length, D = diameter, B = biomass.

**Table S3** Node centrality indices across all treatment networks.

| Treatment | Centrality measurement | Basal  Diam | Veg  TillerN | Veg  TillerL | Veg  TillerD | Veg  TillerB | Rep  TillerN | Rep  TillerL | Rep  TillerD | Rep  TillerB |
| --- | --- | --- | --- | --- | --- | --- | --- | --- | --- | --- |
| NG | Betweenness | 0.427 | 0.427 | -0.970 | 2.174 | -0.621 | -0.621 | -0.272 | -0.970 | 0.427 |
|  | Closeness | -0.051 | 0.020 | -1.109 | 2.224 | -0.938 | -0.729 | 0.537 | -0.224 | 0.270 |
|  | Strength | -0.105 | 0.658 | -1.595 | 1.160 | -0.349 | -0.211 | 0.206 | -1.192 | 1.427 |
|  | Expected Influence | 0.098 | 0.880 | -0.651 | 0.476 | 0.480 | -1.862 | -0.109 | -0.800 | 1.489 |
| CLG | Betweenness | -0.181 | 0.226 | 0.634 | 1.041 | -0.181 | -0.996 | 1.448 | -1.810 | -0.181 |
|  | Closeness | -0.902 | -0.443 | -0.436 | 1.185 | -1.021 | 0.685 | 1.742 | -0.916 | 0.107 |
|  | Strength | -0.956 | -0.069 | -0.690 | -1.276 | 0.140 | 1.228 | 0.591 | -0.616 | 1.649 |
|  | Expected Influence | -0.018 | 0.703 | 0.120 | -0.394 | 0.609 | -1.428 | -0.537 | -0.976 | 1.920 |
| CMG | Betweenness | -0.711 | 0.142 | -0.711 | -0.711 | 1.421 | -0.711 | 0.142 | -0.711 | 1.848 |
|  | Closeness | -0.785 | 0.042 | -0.544 | -1.738 | 0.896 | 0.270 | 0.589 | -0.374 | 1.644 |
|  | Strength | -0.526 | 1.035 | -0.636 | -1.293 | 0.674 | -0.295 | -0.184 | -0.663 | 1.887 |
|  | Expected Influence | -0.526 | 1.035 | -0.636 | -1.293 | 0.674 | -0.295 | -0.184 | -0.663 | 1.887 |
| CHG | Betweenness | -0.685 | 0.105 | -0.843 | -0.369 | 1.528 | -0.843 | 1.687 | -0.843 | 0.264 |
|  | Closeness | -0.253 | 0.435 | -0.232 | -0.196 | 1.269 | -1.390 | 1.749 | -0.472 | -0.911 |
|  | Strength | -0.239 | 1.059 | -1.100 | -0.598 | 1.634 | -0.336 | -0.469 | -1.036 | 1.085 |
|  | Expected Influence | -0.155 | 1.154 | -1.024 | -0.905 | 1.736 | -0.253 | -0.387 | -0.959 | 0.793 |
| WLG | Betweenness | -0.911 | 0.319 | -0.501 | 0.319 | -0.091 | -0.501 | -0.091 | -0.911 | 2.370 |
|  | Closeness | -0.024 | 1.308 | 0.345 | 0.129 | 1.201 | -0.254 | -0.836 | -1.983 | 0.114 |
|  | Strength | -0.418 | 0.504 | -0.313 | 0.413 | 0.924 | 0.021 | -0.516 | -2.038 | 1.424 |
|  | Expected Influence | -0.557 | 0.191 | 0.185 | -1.046 | 1.073 | -0.025 | 0.006 | -1.550 | 1.723 |
| WMG | Betweenness | -0.970 | 0.373 | -0.299 | 0.037 | -0.299 | -0.970 | 1.717 | -0.970 | 1.381 |
|  | Closeness | -1.669 | 0.113 | 0.422 | 0.634 | 0.048 | -1.172 | 1.725 | -0.414 | 0.313 |
|  | Strength | -1.104 | 0.188 | -1.035 | 0.090 | 0.413 | -0.527 | 1.175 | -0.925 | 1.726 |
|  | Expected Influence | -0.713 | 0.079 | -0.485 | -1.141 | 0.887 | -0.616 | 1.191 | -0.835 | 1.633 |
| WHG | Betweenness | -0.943 | 1.037 | -0.377 | -0.094 | -0.377 | -0.943 | -0.094 | -0.377 | 2.168 |
|  | Closeness | -1.034 | 0.186 | -0.208 | 1.439 | -0.919 | -0.766 | 1.250 | -0.920 | 0.971 |
|  | Strength | 0.047 | 0.783 | -1.082 | -0.376 | 0.452 | -0.931 | 0.530 | -1.220 | 1.798 |
|  | Expected Influence | 0.267 | -0.037 | -0.804 | -1.137 | 0.652 | -0.661 | 0.726 | -0.935 | 1.930 |

Note: Values are z-scores (standardized within each treatment network). Blue values indicate strongly positive (z > 0.8); Red values indicate strongly negative (z < −0.8). NG = No-grazing; CLG = Cold-season light grazing; CMG = Cold-season moderate grazing; CHG = Cold-season heavy grazing; WLG = Warm-season light grazing; WMG = Warm-season moderate grazing; WHG = Warm-season heavy grazing; VegTiller = vegetative tiller, RepTiller = reproductive tiller; N = number, L = length, D = diameter, B = biomass.

**Table S4** Key edge weights across grazing treatments.

| Key Edge | NG | CLG | CMG | CHG | WLG | WMG | WHG |
| --- | --- | --- | --- | --- | --- | --- | --- |
| BasalDiam — VegTillerN | **0.506** | **0.476** | **0.440** | **0.498** | **0.423** | **0.430** | **0.550** |
| RepTillerN — RepTillerB | **0.531** | **0.673** | **0.456** | **0.592** | **0.536** | **0.684** | **0.749** |
| VegTillerN — VegTillerB | 0.295 | 0.396 | **0.452** | **0.421** | 0.296 | 0.387 | 0.251 |
| VegTillerL — VegTillerB | 0.156 | **0.532** | 0.328 | 0.368 | 0.230 | 0.295 | 0.267 |
| VegTillerD — RepTillerL | **0.465** | **0.454** | 0.126 | 0.219 | 0.303 | **0.508** | **0.411** |
| BasalDiam — VegTillerD | +0.212 | 0 | 0 | 0 | **-0.107** | **-0.127** | 0 |
| RepTillerL — RepTillerD | 0.181 | 0 | 0.038 | 0 | 0.097 | 0.290 | 0.125 |

Note: Selected edges with ecological significance or notable treatment-dependent variability. Weights are partial correlations estimated by EBICglasso. Bold blue values indicate strong edges (|weight| ≥ 0.4); bold red values indicate negative edges. '0' indicates the edge was set to zero by regularisation (absent from that network). Bootstrap CI analysis confirmed that edges with weight ≥ 0.2 in any treatment generally had 95% CIs excluding zero. BasalDiam–VegTillerD: The sign reversal from positive (NG0: +0.212) to negative (WG4: −0.107; WG8: −0.127) under warm-season grazing is a bootstrap-supported pattern indicating that the association between plant size and vegetative tiller diameter changes direction under warm-season grazing pressure. NG = No-grazing; CLG = Cold-season light grazing; CMG = Cold-season moderate grazing; CHG = Cold-season heavy grazing; WLG = Warm-season light grazing; WMG = Warm-season moderate grazing; WHG = Warm-season heavy grazing; VegTiller = vegetative tiller, RepTiller = reproductive tiller; N = number, L = length, D = diameter, B = biomass.

**
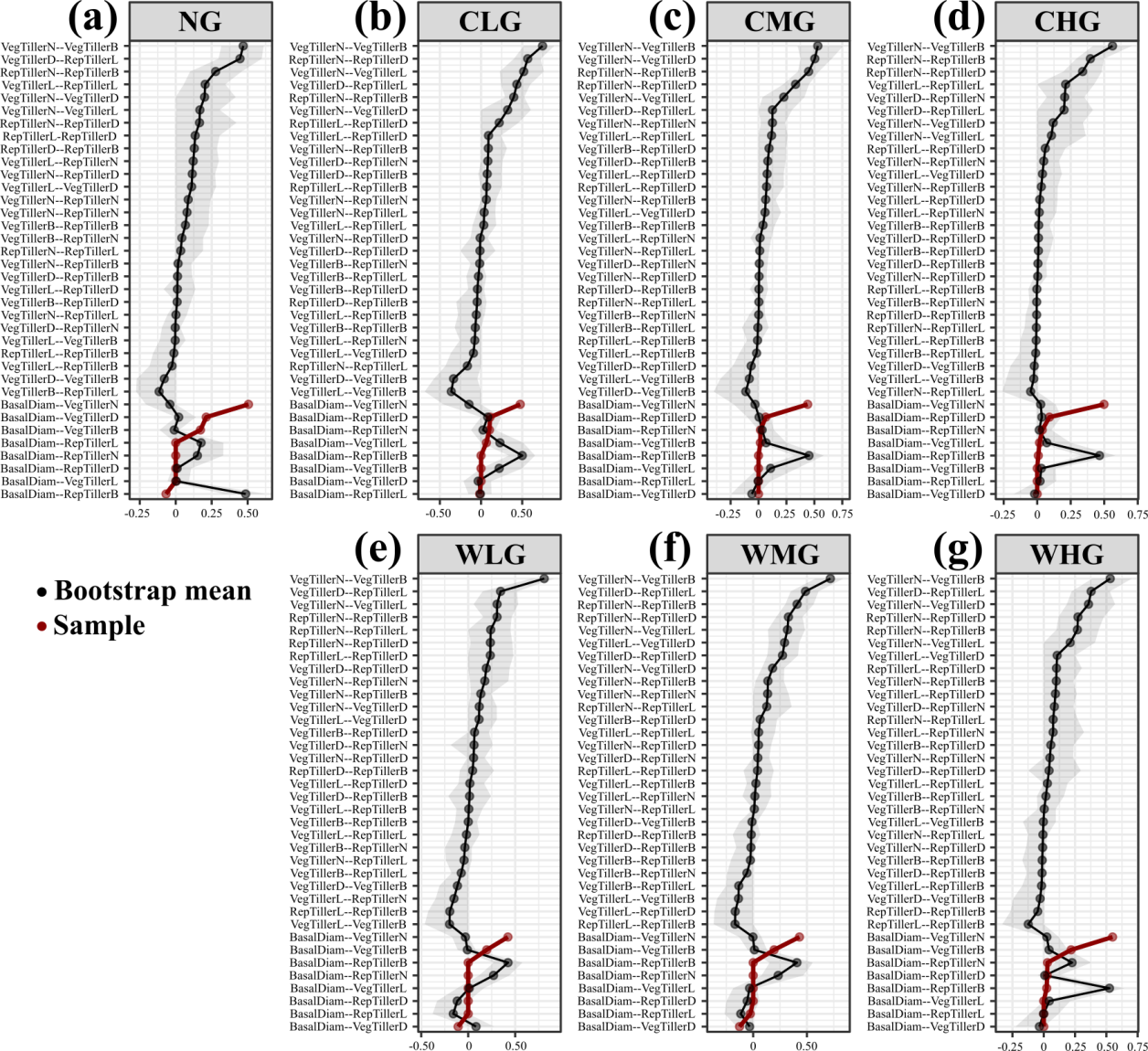
**

**Fig. S1.** Bootstrapped edge-weight confidence intervals. Seven sub-panels (one per treatment) showing bootstrapped 95% confidence intervals (shaded ribbon) and bootstrap means (gray dots) for all estimated edge weights, sorted by estimated weight magnitude. The red dot indicates the observed (non-bootstrapped) edge weight. Edges whose CI ribbon does not cross zero are considered reliably estimated. Note that the dominant edges (RepTillerN–RepTillerB, BasalDiam–VegTillerN) show narrow CIs and large red-dot values, confirming robust estimation, while edges near the regularisation threshold show wider CIs. NG = No-grazing; CLG = Cold-season light grazing; CMG = Cold-season moderate grazing; CHG = Cold-season heavy grazing; WLG = Warm-season light grazing; WMG = Warm-season moderate grazing; WHG = Warm-season heavy grazing; VegTiller = vegetative tiller, RepTiller = reproductive tiller; N = number, L = length, D = diameter, B = biomass.

**
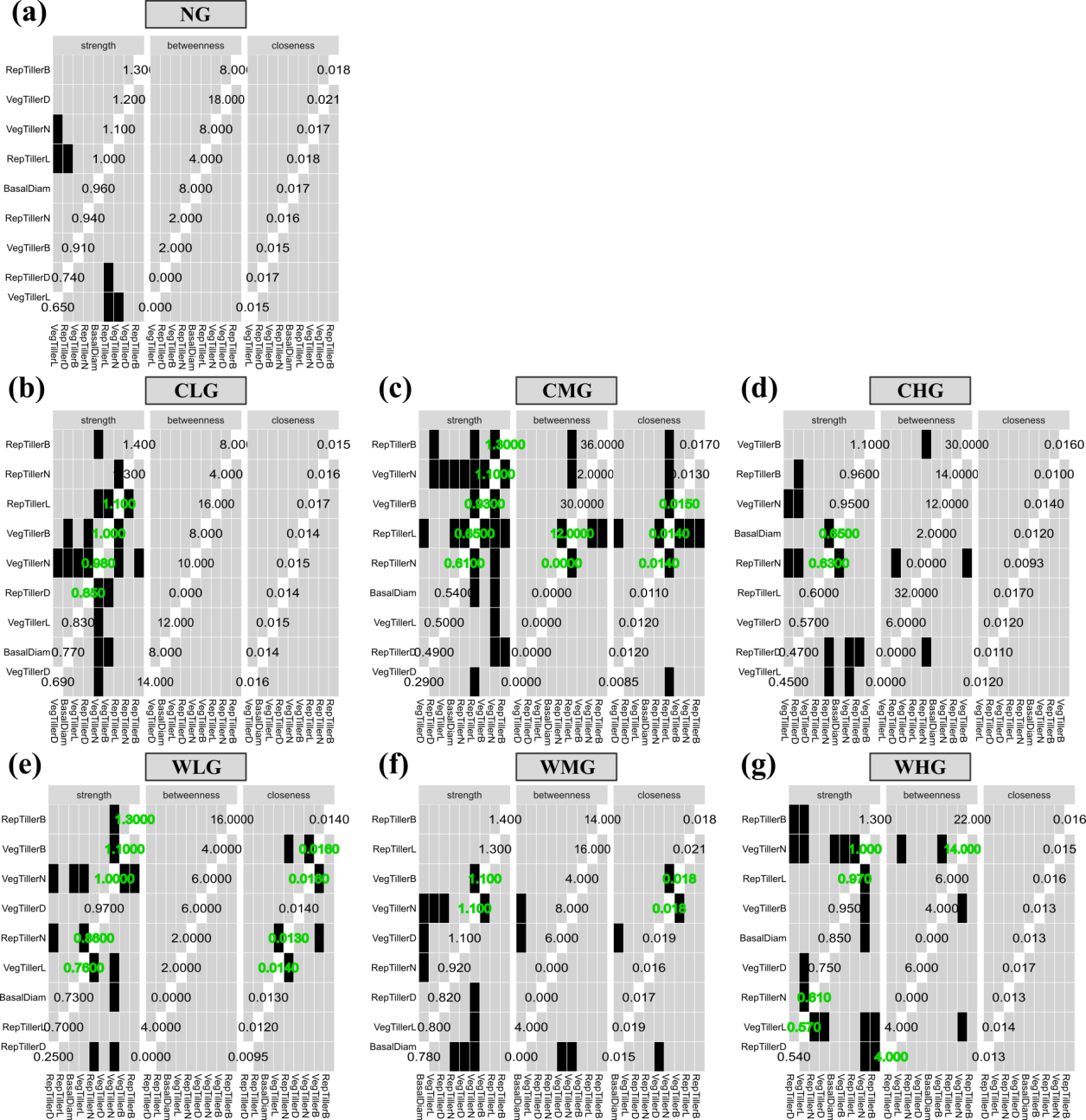
**

**Fig. S2.** Centrality stability heatmaps. Seven heatmap panels (one per treatment) displaying the stability of node centrality rankings (Strength, Betweenness, Closeness) under progressive case-dropping. The y-axis lists nodes sorted by centrality value; the x-axis shows different case-dropping subsets; the x-axis labels show the bootstrap subset IDs. Black cells indicate that the node maintains its ranked position when cases are removed; lighter cells indicate rank instability. NG = No-grazing; CLG = Cold-season light grazing; CMG = Cold-season moderate grazing; CHG = Cold-season heavy grazing; WLG = Warm-season light grazing; WMG = Warm-season moderate grazing; WHG = Warm-season heavy grazing; VegTiller = vegetative tiller, RepTiller = reproductive tiller; N = number, L = length, D = diameter, B = biomass.


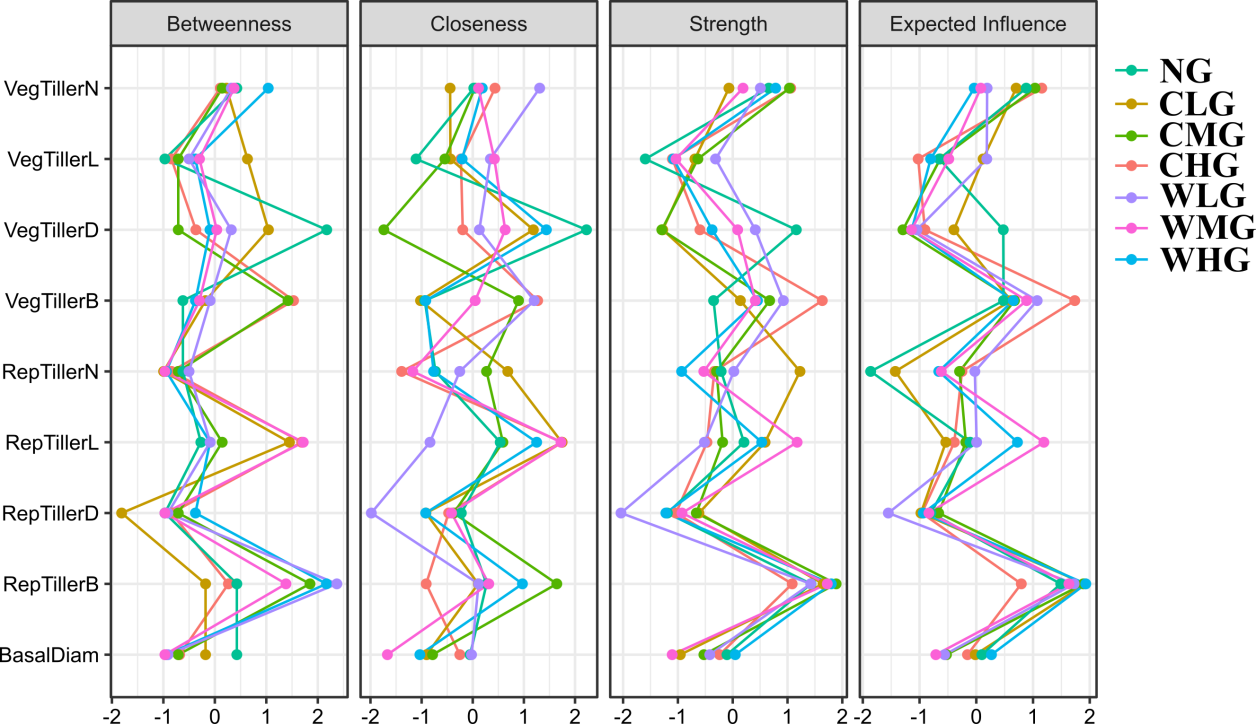


**Fig. S3.** Cross-treatment centrality comparison. A single panel showing standardised centrality indices (Betweenness, Closeness, Strength, Expected Influence) for all nine nodes across all seven treatment networks simultaneously (color-coded by treatment group). This panel facilitates visual comparison of relative centrality shifts associated with grazing season and intensity. RepTillerB consistently shows the highest Strength and Expected Influence across treatments. BasalDiam shows reduced or negative Strength in most grazed treatments compared with the ungrazed reference. NG = No-grazing; CLG = Cold-season light grazing; CMG = Cold-season moderate grazing; CHG = Cold-season heavy grazing; WLG = Warm-season light grazing; WMG = Warm-season moderate grazing; WHG = Warm-season heavy grazing; VegTiller = vegetative tiller, RepTiller = reproductive tiller; N = number, L = length, D = diameter, B = biomass.
